# TFEB Regulates ATP7B Expression to Promote Platinum Chemoresistance in Human Ovarian Cancer Cells

**DOI:** 10.3390/cells11020219

**Published:** 2022-01-10

**Authors:** Raffaella Petruzzelli, Marta Mariniello, Rossella De Cegli, Federico Catalano, Floriana Guida, Elia Di Schiavi, Roman S. Polishchuk

**Affiliations:** 1 Telethon Institute of Genetics and Medicine, 80078 Pozzuoli, Italy; r.petruzzelli@tigem.it (R.P.); decegli@tigem.it (R.D.C.); f.catalano@tigem.it (F.C.); 2 Institute of Physiology, Zürich University, Winterthurerstr. 190, CH-8057 Zürich, Switzerland; marta.mariniello@uzh.ch; 3 Institute of Biosciences and BioResources (IBBR), CNR, 80131 Naples, Italy; elia.dischiavi@ibbr.cnr.it; 4 Department of Neurosciences, Rehabilitation, Ophthalmology, Genetics, Maternal and Child Health (DiNOGMI), University of Genoa, 16132 Genoa, Italy; floriana.guida@edu.unige.it

**Keywords:** ATP7B, TFEB, Golgi, ovarian cancer, chemoresistance

## Abstract

ATP7B is a hepato-specific Golgi-located ATPase, which plays a key role in the regulation of copper (Cu) homeostasis and signaling. In response to elevated Cu levels, ATP7B traffics from the Golgi to endo-lysosomal structures, where it sequesters excess copper and further promotes its excretion to the bile at the apical surface of hepatocytes. In addition to liver, high ATP7B expression has been reported in tumors with elevated resistance to platinum (Pt)-based chemotherapy. Chemoresistance to Pt drugs represents the current major obstacle for the treatment of large cohorts of cancer patients. Although the mechanisms underlying Pt-tolerance are still ambiguous, accumulating evidence suggests that lysosomal sequestration of Pt drugs by ion transporters (including ATP7B) might significantly contribute to drug resistance development. In this context, signaling mechanisms regulating the expression of transporters such as ATP7B are of great importance. Considering this notion, we investigated whether ATP7B expression in Pt-resistant cells might be driven by transcription factor EB (TFEB), a master regulator of lysosomal gene transcription. Using resistant ovarian cancer IGROV-CP20 cells, we found that TFEB directly binds to the predicted coordinated lysosomal expression and regulation (CLEAR) sites in the proximal promoter and first intron region of ATP7B upon Pt exposure. This binding accelerates transcription of luciferase reporters containing ATP7B CLEAR regions, while suppression of TFEB inhibits ATP7B expression and stimulates cisplatin toxicity in resistant cells. Thus, these data have uncovered a Pt-dependent transcriptional mechanism that contributes to cancer chemoresistance and might be further explored for therapeutic purposes.

## 1. Introduction

The Golgi complex represents one of the key compartments in the regulation of copper (Cu) homeostasis [1]. Cu is an indispensable micronutrient, which supports the activities of several vitally-important enzymes [2] and regulates the activities of signaling networks implicated in cell proliferation, autophagy, and lipid metabolism [3,4,5]. The ability of the Golgi to control intracellular Cu levels relies on the activities of two highly homologous Cu-transporting ATPases, ATP7A and ATP7B, which normally load Cu onto newly-synthetized Cu-dependent proteins moving across the Golgi compartment [1]. In response to elevated Cu, the Golgi exports ATP7A/B towards specific post-Golgi sites, where these Cu ATPases drive sequestration/excretion of excess metal, thereby protecting cells from its toxicity [1]. The importance of this process is underscored by fatal Wilson disease, which is caused by the toxic accumulation of copper in the liver due to loss of ATP7B function [6].

Although the ability of ATP7B to counteract metal toxicity is essential for the homeostasis of liver tissue, such ATP7B ability might play a negative role in cancer. A growing body of evidence indicates that ATP7B is used by malignant cells to detoxify platinum (Pt)-based drugs, thereby promoting tumor resistance to chemotherapy [7,8,9,10,11,12]. Cisplatin and similar Pt drugs have demonstrated excellent efficiency in the treatment of different solid tumors, including ovarian, lung, head and neck, and gastric cancers [13,14]. However, the final outcome of these agents is often hampered by the development of drug resistance which is still a significant barrier to successful treatment in the clinic [13,14,15]. Indeed, enhanced DNA repair, increased detoxification, or modulation of Pt uptake and/or efflux can cause decreased drug accumulation leading to cisplatin resistance [9].

Recent studies suggest that the transporters that mediate Cu homeostasis may also regulate the intake and excretion of Pt agents, thereby modulating the cytotoxic activity of these drugs [7,10,16]. In particular, ATP7B has been suggested to play a significant role in this process. Physiologically, in response to Cu, ATP7B translocates from the Golgi to lysosomes, where it facilitates Cu sequestration and clearance from the cells [17]. Similar to Cu, Pt was shown to undergo ATP-dependent sequestration to lysosomes [18,19], suggesting that both metals might share similar mechanisms of acquisition, distribution, and elimination. Indeed, suppression of ATP7B export from the Golgi significantly promotes cisplatin toxicity [12], while overexpression of ATP7B in tumors results in a negative response to Pt chemotherapy [8,16,20]. In this regard, Pt-dependent mechanisms that regulate ATP7B expression in tumor cells are of great importance and have to be better understood.

To address this challenging task, we considered a growing body of evidence that links ATP7B to the endo-lysosomal compartment [21,22]. Apparently, a lower lysosomal pH might stimulate ATP7B catalytic activity and, therefore, favor ATP7B-mediated sequestration of Pt [23]. This mechanism (together with several others) enables the lysosome to operate as a main player in anti-cancer drug resistance [11,24]. Thus, to tolerate incoming drugs, the tumor cells have to activate lysosomal biogenesis, which might be driven by TFEB, a master regulator of lysosomal gene network [25]. Indeed, recent publications suggest that TFEB is involved in Pt resistance in some malignant cells [26] and could be activated in response to Cu and similar toxic metals [26,27]. These findings prompted us to investigate whether transcription of ATP7B in Pt-resistant tumor cells is regulated by TFEB. We found that the promoter region of *ATP7B* contains CLEAR binding sites, which are used by TFEB for Pt-dependent activation of ATP7B transcription in resistant ovarian cancer cells. Correspondingly, TFEB suppression results in reduced ATP7B expression and elevated susceptibility of tumor cells to cisplatin. Our data suggest that this TFEB transcriptional connection with ATP7B could play a particularly important role in the development of Pt resistance and might be explored to improve the efficiency of Pt-based chemotherapy. 

## 2. Materials and Methods

### 2.1. Cell Culture and Transfection

Cisplatin-sensitive human ovarian cancer IGROV and A2780 cells and the corresponding cisplatin-resistant lines IGROV-CP20 and A2780-CP20 were obtained from Dr. A. Sood (University of Texas, MD Anderson Cancer Center). The cells were grown in RPMI supplemented with 15% FBS, 2 mM L-glutamine, and 1% penicillin and streptomycin. Cell treatments were performed using *cis*-Diammineplatinum (II) dichloride (cisplatin) (Cat# P4394) from Sigma. For small interfering RNAs (siRNA) experiments, KO of TFEB was achieved using siRNA purchased from Dharmacon (Lafayette, CO, USA). Scrambled siRNAs were used as negative controls. IGROV-CP20 cells were transfected with siRNA using Lipofectamine RNAiMAX reagent (Invitrogen, Carlsbad, CA, USA). After 48 h of interference, the cells were treated with 50 μM cisplatin for 24 h. Overexpression of TFEB was performed transfecting IGROV-CP20 cells with 1ug of TFEB3x flag obtained from Dr. A Ballabio (TIGEM, Pozzuoli, Italy) and using Mirus (Madison, WI, USA) as transfection reagent. Adenoviruses carrying cDNA of ATP7B-WT-GFP [17] were used at 10 of Multiplicity of Infection (MOI) to transduce IGROV-CP20 cells for 24 h.

### 2.2. MTT Cell Viability Assay

The viability of IGROV or IGROV-CP20 cells was determined by measuring their ability to reduce the tetrazolium salt (MTT) to a formazan as described in [12]. The results were normalized to the absorbance value in untreated cells (considered to be 100% viable).

### 2.3. Immunofluorescence

Immunofluorescence experiments were performed on control and treated IGROV and IGROV-CP20. Briefly, cells underwent a 10-min slide fixation with 4% paraformaldehyde in 0.2 M HEPES buffer, followed by incubation with blocking-permeabilization solution (0.5% BSA, 0.1% saponin, and NH4Cl 50 mM in PBS) for 30 min, before the addition of primary antibodies. Primary and secondary antibodies were diluted in blocking permeabilization solution and added to the cells for 1 h or 45 min, respectively. Primary antibodies used were: mouse anti-human TFEB monoclonal Ab (MyBiosource, San Diego, CA, USA), rabbit anti-human ATP7B (Abcam, Cambridge, UK), mouse anti-human CD63 (Santa Cruz Biotechnology, Santa Cruz, CA, USA), Secondary Alexa Fluor 488, 568 conjugated antibodies for immunofluorescence were from Invitrogen-Life Technologies (Grand Island, NE, USA). Samples were examined under a confocal microscope (ZEISS LSM 700; Carl Zeiss AG, Jena, Germany). 

### 2.4. DNA Adduct Evaluation by Dot Blot

To detect the amount of DNA adducts in the control and TFEB silenced IGROV-CP20 cells, DNA was isolated using the Quick-DNA Miniprep Plus Kit (Zymo Research, Irvine, CA, USA) and 1 ug DNA was spotted on a piece of Nytran N nylon blotting membrane (GE Healthcare Life Sciences, Boston, MA, USA). Dot blot experiments were performed using an ant adduct antibody (ICR4, Merck, Darmstadt, Germany) following the previously-described procedure [12].

### 2.5. Quantitative Real-Time PCR (RT-qPCR)

To evaluate the mRNA expression levels of TFEB and ATP7B, total RNA was isolated using the RNeasy Protect Mini kit (Qiagen, Germantown, MD, USA) from treated and control cells before reverse transcription of the RNAs using QuantiTect Reverse Transcription kits (Qiagen). RT-qPCR experiments were performed using Light Cycler 480 Syber Green I Master Mix (Roche, Basel, Switzerland) using a LightCycler 480 II Real-Time System (Roche) in 96-well plates. RT-PCR results were analyzed using the 2^−ΔΔCt^ method, normalized against the housekeeping gene β-actin. The following primers were used:

β-ACTIN forward: (5′-AAGAGCTACGAGCTGCCTGA-3′);
β-ACTIN reverse: (5′-GACTCCATGCCCAGGAAGG-3′);
ATP7B forward: (5′-TCTCTGGTCATCCTGGTGGTT-3′);
ATP7B reverse: (5′-GGGCTTCTGAGGTTTTGCTCT-3′);
TFEB forward: (5′-CAAGGCCAATGACCTGGAC-3′);
TFEB reverse: (5′-AGCTCCCTGGACTTTTGCAG-3′)

### 2.6. Western Blotting

Proteins were extracted with Lysis Buffer (0.5% Triton X-100, 20 mM Tris/HCl (pH 7.4), 150 mM NaCl, 1mM EDTA (pH: 8), 0.5% NP-40, 10% glycerol, supplemented with 1× protease inhibitor cocktail (Sigma, St. Louis, MO, USA) and boiled for denaturation. Sodium dodecyl sulphate-polyacrylamide gel electrophoresis (SDS-PAGE) was performed on samples containing 30 ug of protein using 4–15% pre-cast polyacrylamide gel (Biorad, Hercules, CA, USA). After electron transfer and blocking with 1% BSA in PBS, the membranes were incubated overnight at 4 °C with primary antibodies. Primary antibodies used: rabbit anti-human antibodies against ATP7B (1:1000, Cat#124973) was from Abcam, mouse GAPDH (1:2000, Cat# sc32233) was from Santa Cruz. After incubating with anti-rabbit or anti-mouse secondary antibodies for 60 min at room temperature, filters were incubated with chemiluminescent substrate Pierce ECL Western Blotting Substrate (Cat #32106, Thermo Scientific, Waltham, MA, USA) according to the manufacturer’s instructions. 

### 2.7. Chromatin Immunoprecipitation Assays (ChIP)

ChIP assays were performed as previously described [28] using the following antibodies: TFEB (Cell signaling, Danvers, MA, USA) and rabbit IgG (Merck). Prior to chromatin recovery, IGROV-CP20 cells were treated with 50 uM cisplatin for 24 h. DNA recovered from ChIP was amplified with RT q-PCR using the following primers spanning the predicted CLEAR sites:

ATP6V1H Forward: (5′-TCGGGAATCCAGTTGTCCG-3′);
ATP6V1H Reverse: (5′-GCCGCACAGGTAGAAGGAA-3′);
ChIP3 Forward: (5′-GCGTGAGATCCCAGTACAGTGT-3′);
ChIP3 Reverse: (5′-ACAATGTCCTCTGCCGTGCC-3′);
ChIP4 Forward: (5′-GCGCAACTTTGAATCATCCGT-3′);
ChIP4 Reverse: (5′-TAAAGCAAACAGGGGTCCGG-3′);
ChIP5/6 Forward: (5′-CAGGCGCAGAGTCCGAGGAG-3′);
ChIP6 Reverse: (5′-CTGTACTGGGATCTCACGCG-3′);
ChIP8 Forward: (5′-CCAAATGAAGGGGCGGTTCC-3′);
ChIP8 Reverse: (5′-GAGGAGGCGCAGAGTGTGAG-3′);
ChIP10 Forward: (5′-CCAGGTGACCTTTTGCTCTGA-3′);
ChIP10 Reverse: (5′-TGGCTGTGATCTGTCTCTCCT-3′);
ChIP11 Forward: (5′-ATGTCTTGGCGTGGGAGAAAA-3′);
ChiP11 Reverse: (5′-ATGAATTTTGAGGTGCGGGGT-3′);
ChIP12 Forward: (5′-CAGCTCCAGGGATGTCTTGG-3′);
ChIP12 Reverse: (5′-TCTCCAGCATCAGACCCCTT-3′).

Fold enrichment are reported relative to normal IgG control for each sample. Data are represented as a mean of three different experiments. 

### 2.8. Bioinformatic Prediction of TFEB Binding Sites

To bioinformatically identify the CLEAR motifs in the ATP7B promoter, we used the TFEBdb (http://tfeb.tigem.it, accessed on 10 November 2021 [29]). The ATP7B promoter sequence included 2000 bp upstream and 500 bp downstream from the transcription start site (TSS) for the human isoform of ATP7B. The ATP7B sequence was downloaded from UCSC Genome Browser website [30] using the hg19 genome version. The position-weight matrix of the TFEB consensus binding sequence was identified using the Weeder tool 2.0, as previously described [31]. For the determination of the binding sites, the package match-PWM by Biostrings with default parameters was applied as described in De Cegli et al. [29]. The threshold for the PWM Score (minimal score) was set to 0.85.

### 2.9. Vector Constructs and Reporter Assays

A PLX304 vector containing the TFEB (NCBI Reference Sequence: NM_001271944) gene was kindly provided by Dr. Carmine Settembre (TIGEM). A region of 1000 bp from 538 upstream to 484 downstream from the TSS of ATP7B, including all three predicted CLEAR sites, was amplified with specific primers that contained XhoI and HINDIII enzyme-specific restriction sites:

ATP7B full (XhoI)Forward: (5′CTAGCCCGGGCTCGAGCGTGGCCTGTGATTGACAGC-3′);
ATP7B full (HINDIII) Reverse: (5′-CCGGAATGCCAAGCTTTTAGGGCTCTAGCACAGCAACTCG-3′).

The product was cloned into a TOPO vector Blunt (Invitrogen), and removed using XhoI/HINDIII sites in a pGL4 Basic vector (Promega, Madison, WI, USA). This construct was named ATP7B prom. With the same methodology, another three expression vectors were generated, each including only one CLEAR site using the following primers:

ATP7B (CLEAR 1 XhoI) Forward: (5′-CTCGAGGCGATCGATTTTCCAGGTGCGGA-3′);
ATP7B (CLEAR1 HINDIII) Reverse: (5′-TTCGAA CAGTGCCACAATGTCCTCTG-3′);
ATP7B (CLEAR2 XhoI) Forward: (5′-CTCGAGC GCGCGCAACTTTGAATCATC-3′);
ATP7B (CLEAR2 HINDIII) Reverse: (5′-TTCGAATGGCTGTGATCTGTCTCTCC-3′);
ATP7B (CLEAR3 XhoI) Forward: (5′-CTCGAGGC GCCAGTCGGAAAGTGAGTTT-3′);
ATP7B (CLEAR3 HINDIII) Reverse: (5′-TTCGAATTAGGGCTCTAGCACAGCAAC-3′).

After TOPO cloning, each fragment was subcloned into pGL4 Basic vector using XhoI/HINDIII sites, and the resulting constructs were named: CLEAR1, CLEAR2 and CLEAR3, respectively. IGROV-CP20 were seeded in 12-well plates (3 × 10^4^ cell/well) and transiently transfected using Lipo293 transfection reagent (Signagen, Frederick, MD, USA) following the manufacturer’s procedure. PGL4 and PLX304 empty vectors were co-transfected to check PLX304-TFEB activity on the CLEAR sites within the ATP7B sequences harbored by the PGL3-ATP7B different constructs. Luciferase activity was measured using the dual luciferase reporter assay system (Promega) with a GloMax 20/20 luminometer (Promega). Normalization was performed using a PLTK-Renilla luciferase vector (Promega).

### 2.10. Statistical Analysis

Statistical analysis was performed using GraphPad Prism 8.0.2 Software. Data are expressed as mean ± standard deviation (or standard error where indicated) and were collected from multiple independent experiments performed on different days. Statistical significance for all data was computed using the Student *t*-test or one-way ANOVA (for all figures, * *p* < 0.05, ** *p* < 0.01, and *** *p* < 0.001 indicate statistical significance).

## 3. Results

### 3.1. TFEB Regulates ATP7B Expression in Pt-Resistant Ovarian Cancer Cells

**Figure 1:**
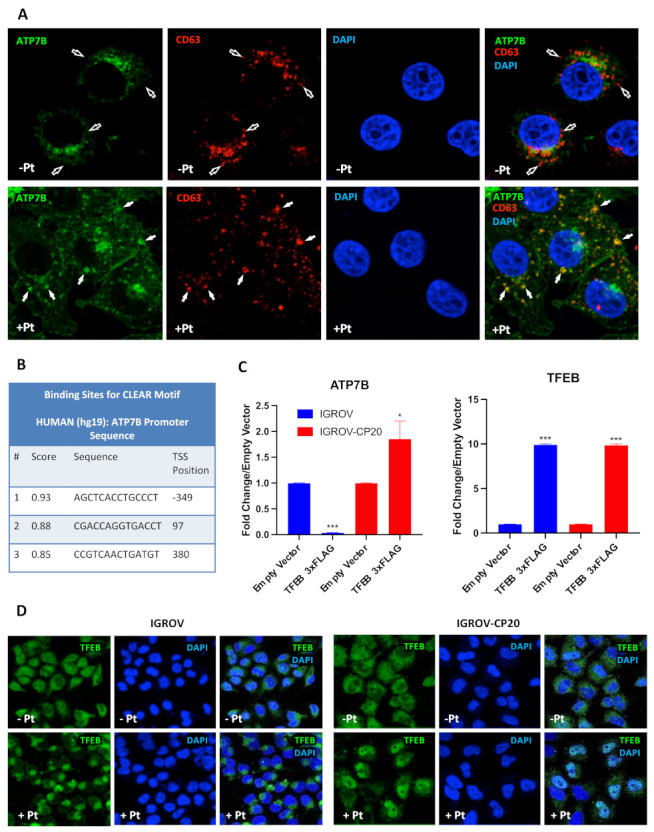
ATP7B and TFEB expression and localization in Pt-resistant cells. (**A**) IGROV-CP20 cells expressing ATP7B-GFP were fixed for confocal microscopy directly (Pt−) or after 4 h incubation with 50 µM cisplatin (Pt+). Images show colocalization of CD63 with ATP7B in late endosomal/lysosomal organelles (filled arrows) in Pt-treated cells, while CD63-positive structures in untreated cells did not contain ATP7B (empty arrows). (**B**) The Table shows predicted CLEAR motifs (see methods) in the transcription start site (TSS) ± 500 base region of ATP7B that were mapped to −349, +97, and +380 positions. (**C**) Expression levels of *ATP7B* or *TFEB* in Pt-sensitive IGROV and Pt-resistant IGROV-CP20 cells upon overexpression of a TFEB-3×FLAG vector. RT-qPCR shows that *ATP7B* mRNA is upregulated in IGROV-CP20 cells upon TFEB overexpression compared to the control cells expressing empty vector (***, *p* < 0.001; *, *p* < 0.1, *t*-test; *n* = 3 experiments). An equal amount of TFEB expression is reached upon overexpression of a TFEB-3xFLAG in both cell lines (***, *p* < 0.001, *t*-test compared to control cells expressing empty vector; *n* = 3 experiments). (**D**) IGROV and IGROV-CP20 cells were fixed for confocal microscopy directly (Pt−) or after 24 h incubation with 50 µM cisplatin (Pt+)**.** Representative confocal microscopy images show that treatment with cisplatin stimulated translocation of TFEB into the nucleus in resistant IGROV-CP20 cells but not in parental IGROV cells.

The transcription of numerous lysosomal genes is regulated by TFEB that, in response to various stimuli translocates to the nucleus, where it activates the transcription of lysosomal and autophagic genes [25,32,33]. We reasoned that a cohort of TFEB target genes might include ATP7B, whose elevated expression confers Pt-resistance in tumor cells. The association between ATP7B and the late-endosomal/lysosomal compartment has previously been documented in different studies [22,34,35], while pilot experiments in resistant IGROV-CP20 cells have revealed ATP7B relocation to LAMP1-positive structures in response to cisplatin (Figure 1A). This prompted us to investigate whether the *ATP7B* promoter contains CLEAR motifs, which are used by TFEB to activate the transcription of lysosomal genes [32,33]. Therefore, we employed the TFEBdb tool [29] to search for CLEAR sequences in the −2000 to +500 base region with respect to the *ATP7B* transcription start site (TSS). Bioinformatic analysis revealed the presence of 3 putative CLEAR sites located in positions −349 (CLEAR1), +97 (CLEAR2), and +380 (CLEAR3) from the *ATP7B* TSS (Figure 1B), indicating that *ATP7B* transcription might be regulated by TFEB. To test this hypothesis, we initially employed a widely-used TFEB overexpression approach [32]. TFEB was transfected into cisplatin-sensitive parental IGROV cells and a resistant IGROV-CP20 cell line, and the expression of ATP7B was evaluated by qRT-PCR. Figure 1C shows that overexpression of TFEB significantly increased the expression of ATP7B in the resistant IGROV-CP20 cells but not in the parental sensitive line. This suggests that the ability to activate *ATP7B* transcription in resistant cells does not depend on TFEB expression levels and might be defined by different regulation of its activity in Pt-resistant and sensitive cells. To test this possibility, we then investigated the behavior of TFEB in response to cisplatin. Confocal microscopy revealed a Pt-induced translocation of TFEB from the cytoplasm to the nucleus in IGROV-CP20, while in parental IGROV cells TFEB remained in the cytoplasm regardless of cisplatin presence (Figure 1D). These findings suggest that the ability of TFEB to regulate ATP7B expression in resistant cells relies on Pt-induced nuclear translocation of this transcription factor, while in Pt-sensitive cells, this regulatory mechanism does not work. This difference is also mirrored by higher ATP7B expression in IGROV-CP20 cells, as previously reported in our previous study [12].

### 3.2. ATP7B Gene Is a Direct Target of TFEB

**Figure 2:**
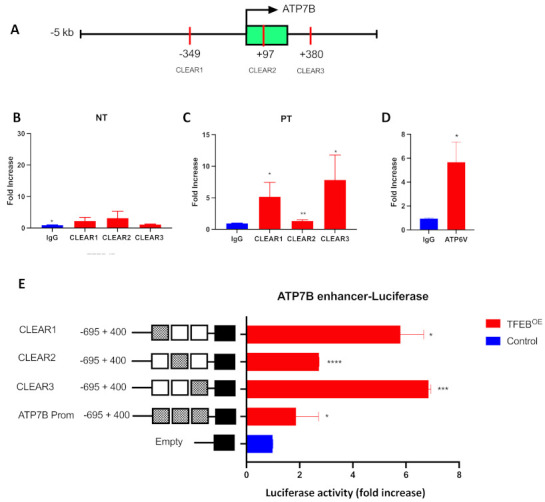
TFEB binds to ATP7B and activates its transcription in a Pt-dependent manner. (**A**) Schematic representation of the ATP7B proximal promoter (−5 kb from TSS) and first intron (+380 from TSS) where the CLEAR sites have been predicted with relative positions from the TSS. (**B**,**C**) Chromatin was isolated from IGROV-CP20 in untreated (NT) conditions (**B**) or upon 24 h exposure to 50 µM Pt (**C**) and subjected to ChIP with antibody against endogenous TFEB or control IgG. PCR amplification revealed that TFEB-immunoprecipitated chromatin exhibited enrichment in *ATP7B* CLEAR sites in Pt-treated cells. DNA enrichment is expressed as fold increase compared to the control IgG (**, *p* < 0.01; *, *p* < 0.05, *t*-test; *n* = 3 experiments). (**D**) Amplification of bona fide TFEB target *ATP6V* from immunoprecipitated chromatin was used as positive control for the ChiP experiment (*, *p* < 0.05; t-test; *n* = 3 experiments). (**E**) The luciferase reporter constructs harboring the 3 CLEAR sites driven by the ATP7B promoter showed a significant increase in luciferase activity upon overexpression with a PLX3-TFEB (TFEB^OE^) compared to the controls in cells treated with Pt. Data are represented as mean ± SD (****, *p* < 0.0001; ***, *p* < 0.001, *, *p* < 0.05, *t*-test; *n* = 3 experiments).

Although the above findings suggest that TFEB regulates ATP7B expression in resistant cells, it remains unclear whether TFEB promotes *ATP7B* transcription directly by binding to its CLEAR motifs or employing a different regulatory mechanism. To discriminate between these scenarios, we subjected IGROV-CP20 cells to a chromatin immunoprecipitation (CHIP) procedure that allows us to directly assess the binding specificity of TFEB to the CLEAR sites of its target genes [32,33]. The chromatin of resistant IGROV-CP20 cells was immunoprecipitated with a TFEB-specific antibody with or without the addition of Pt. PCR amplification of 3 the putative CLEAR sites (Figure 2A) revealed a specific 15-fold increase of TFEB binding to the CLEAR motif situated in the proximal promoter (−349), a 10-fold binding increase to the CLEAR motif located in the first intron (+380), and a 2-fold increase to the CLEAR motif within the first exon when the chromatin of resistant cells was treated with Pt (Figure 2B,C) compared to the unspecific IgG binding. No significant enrichment was observed in the untreated chromatin, which indicates the specificity of the interaction upon Pt exposure (Figure 2B). To control the specificity and efficiency of CHIP, we tested whether TFEB binds the CLEAR site of known TFEB target *ATP6V* [32,33]. As expected, PCR amplification revealed that *ATP6V* showed significant chromatin enrichment when immunoprecipitated with a TFEB antibody (Figure 2D). These data indicate that TFEB directly binds 3 CLEAR sites within *ATP7B* only upon Pt addition, suggesting a transcriptional connection that is driven by Pt resistance.

To further understand whether direct binding of TFEB to the *ATP7B* CLEAR sites is able to activate transcriptional processes, a standard luciferase reporter assay [32] was performed in IGROV-CP20 cells. A region of 1000 bp from −538 to +484, with respect to the ATP7B transcription start site, which includes all the 3 CLEAR motifs, has been cloned into a PGL3 vector. We also generated 3 PGL3 vectors containing all the single CLEAR sites found in the ATP7B promoter, as described in the method section. As shown in Figure 2E, a significant promoter-driven increase in transcription was observed when the cells were co-transfected with the constructs harboring the single CLEAR sites (CLEAR1 at −349, CLEAR2 at +97, and CLEAR3 at +380) and a TFEB expression vector compared to the control upon Pt exposure. A moderate (but significant) increase in luciferase activity was found when we co-transfected the construct harboring all the CLEAR motifs with the TFEB expression vector compared to the empty vector used as a control (Figure 2E). These data confirm that TFEB is functionally responsible for *ATP7B* transcription through direct binding to the CLEAR motif within the upstream proximal promoter.

Interestingly, the construct harboring all 3 *ATP7B* CLEAR sites appeared to be less efficient in increasing transcription of luciferase reporter compared to the constructs containing single CLEAR sites (Figure 2E). This might be due to the effect of the so-called “homotypic cluster”, a group of adjacent binding sites for the same transcription factor [36]. Such homotypic clusters usually reside in bidirectional promoters, like this of *ATP7B*, which shares more than 1 kb bidirectional promoter with the *ALG11* gene [37] and might slow down the kinetics of the transcription process compared to a promotor region with a single binding site [36]. 

### 3.3. TFEB Stimulates ATP7B Expression in a Pt-Dependent Manner

**Figure 3:**
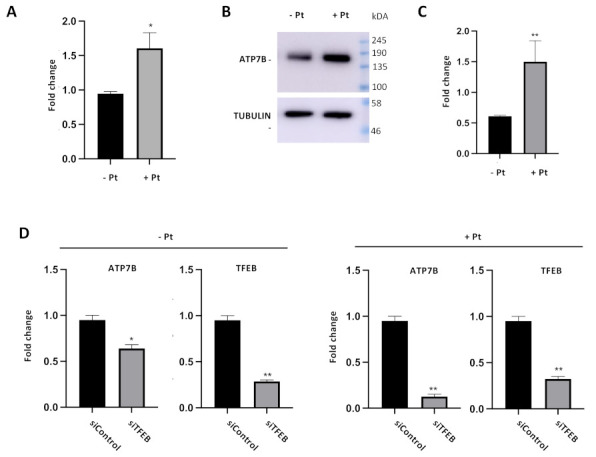
Cisplatin and TFEB impact on ATP7B expression in resistant cells. (**A**–**C**) ATP7B mRNA (**A**) and protein levels (**B** and quantification in **C**) were increased in IGROV-CP20 cells treated with 50 uM Pt compared to the untreated condition (**, *p* < 0.01; *, *p* < 0.05, *t*-test; *n* = 3 experiments). (**D**) RT-qPCR analysis shows a decrease in ATP7B expression levels upon silencing of TFEB (siTFEB) compared to scramble siRNA control (siControl) (**, *p* < 0.01; *, *p* < 0.05, *t*-test; *n* = 3 experiments).

Our CHIP and luciferase reporter experiments clearly suggest that Pt stimulates TFEB binding to the *ATP7B* promoter and further transactivation of *ATP7B* in resistant cells. We reasoned that this should result in elevated ATP7B expression in response to cisplatin. To test whether this is the case, mRNA levels and protein levels of ATP7B were assessed by qRT-PCR and by Western blot, respectively. Figure 3A, B demonstrates that both mRNA and protein levels of ATP7B increased in Pt-treated cells. Further, we investigated whether this Pt-dependent increase in ATP7B expression relies on TFEB activity. To this end, we silenced TFEB in control and Pt-treated cells and assessed the impact of the silencing on ATP7B expression. QRT-PCR revealed that TFEB depletion in control IGROV-CP20 cells only moderately reduces ATP7B expression (Figure 3C). On the contrary, Pt-treated IGROV cells exhibited a significantly higher degree of ATP7B downregulation upon TFEB knockdown (Figure 3D). Taken together, these findings indicate that exposure to Pt activates TFEB-mediated expression of ATP7B.

### 3.4. TFEB Suppression Promotes Cisplatin Toxicity in Resistant Ovarian Cancer Cells

**Figure 4:**
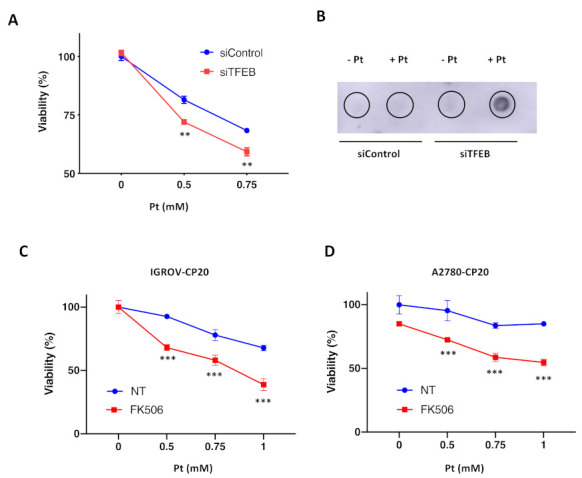
TFEB suppression promotes cisplatin toxicity in resistant ovarian cancer cells. (**A**) TFEB-silenced (siTFEB) and control (siControl) IGROV-CP20 cells were exposed to different concentrations of cisplatin (Pt) for 24 h and then subjected to MTT assay to evaluate their viability. Silencing of TFEB results in increased IGROV-CP20 cell mortality upon different Pt concentrations (**, *p* < 0.01, ANOVA; *n* = 3 experiments). (**B**) DNAs from TFEB-silenced (siTFEB) and control (siControl) IGROV-CP20 cells were isolated directly (−Pt) or 24 h after treatment with 0.5 mM cisplatin (+Pt) and analyzed using dot blot with an antibody that recognizes Pt-DNA adducts. The analysis revealed an increased amount of DNA adducts in TFEB-depleted IGROV-CP20 cells. (**C**,**D**) IGROV-CP20 (**C**) and A2780-CP20 (**D**) resistant cells were treated with 10 µM FK506 and then exposed to increasing concentrations of cisplatin (Pt). MTT assay shows that FK506 promoted Pt toxicity in both cell lines (***, *p* < 0.001, ANOVA; *n* = 3 experiments).

Our previous studies suggest that ATP7B provides a significant contribution to Pt-tolerance that was observed in tumor cells of ovarian origin [12]. Considering our new findings that TFEB promotes ATP7B expression, TFEB targeting is expected to circumvent Pt drug resistance in these cells. To test whether this is the case, we evaluated the impact of TFEB silencing on cisplatin toxicity in IGROV-CP20 cells. MTT-based cell viability assay revealed that TFEB depletion significantly reduced tolerance of IGROV-CP20 cell to cisplatin (Figure 4A). The main toxicity mechanism of Pt-based drugs requires the formation of Pt-DNA adducts, which trigger apoptosis [14]. Therefore, we employed a DNA dot blot approach [12] to test whether DNA adduct formation is stimulated by TFEB depletion. We found that DNA adducts were really scarce in control IGROV-CP20 cells exposed to Pt, while TFEB-silenced cells exhibited a significant increase in the amount of DNA adducts (Figure 4B), which correlated with elevated Pt toxicity in these cells (Figure 4A).

In addition to RNAi, we decided to inhibit TFEB using an alternative pharmacological approach. Previous studies indicated that nuclear translocation of TFEB requires its dephosphorylation by calcineurin phosphatase [38]. Specific calcineurin inhibitor FK506 has been shown to block TFEB translocation to the nucleus, thereby inhibiting the transactivation of downstream genes [38]. Thus, control and FK506-treated IGROV cells were exposed to cisplatin, and their viability was assessed with an MTT assay. We found that FK506 significantly promoted Pt toxicity in resistant IGROP-CP20 cells (Figure 4C), indicating that nuclear translocation of TFEB is required for resistance. 

Finally, we tested whether this TFEB suppression strategy is effective in another ovarian cancer A2780-CP20 cell line, whose resistance to cisplatin relies on ATP7B in a significant manner [12,39]. Considering the feasibility of FK506 use, we employed this inhibitor to assess how the block of TFEB nuclear translocation impacts cisplatin toxicity in A2780-CP20 cells. MTT assay revealed that also in this resistant cell line FK506 was capable of promoting Pt-mediated cell death (Figure 4D). This indicates that the ability of TFEB to upregulate ATP7B expression in response to cisplatin might contribute to the chemoresistance of different ovarian tumor cell lines.

## 4. Discussion

Understanding the signaling mechanism that controls ATP7B expression represents an important issue due to its extremely important ability to counteract the toxicity of transition metals such as copper, silver, and platinum [6,9,10,40]. This property of ATP7B is particularly relevant in oncology because both elevated ATP7B expression levels and its post-Golgi trafficking have been shown to correlate with tumor cell resistance to Pt-based chemotherapy [7,8,12,16,20]. In this context, our findings shed light on a new transcription mechanism that regulates ATP7B expression in resistant tumor cells through Pt-dependent activation of TFEB. Indeed, in our study, we found that transcriptional activation of *ATP7B* was regulated by TFEB only in resistant IGROV-CP20 cells while no correlation was found in the sensitive counterpart, meaning that the transcriptional link between TFEB and ATP7B is specific for the chemoresistant phenotype. Using ChIP and functional Luciferase Assays, we confirmed that TFEB is able to directly transactivate *ATP7B* in the presence of Pt in resistant ovarian cancer cells. These results revealed a control system by which TFEB coordinates the trans-Golgi protein with the lysosomal resistance pathways to protect ovarian cancer from the active Pt molecules.

We would like to underscore that this TFEB-mediated transcriptional mechanism is activated in a Pt-dependent manner. This poses a question, namely, what are the mechanisms that allow Pt to modulate TFEB activity in tumor cells? We found that cisplatin triggers TFEB translocation from the cytoplasm to the nucleus in resistant IGROV-CP20 cells, but not in parental sensitive cells. Such translocation has been reported to happen in response to the lysosomal sequestration of drugs, including Pt-based drugs [24,26]. The presence of these xenobiotic substances in the lysosome interior may cause generic lysosomal stress, which usually results in TFEB activation and relocation to the nucleus [24,32,41]. Comparison of proteomes from resistant and sensitive tumor cells suggests that abundant expression of several lysosomal proteins allows resistant cells to better sequester cisplatin in the lysosomal compartment. These lysosomal proteins include ATP7B itself and V-ATPases, which promote lysosome acidification and, thereby, stimulate ATP7B activity [23,42]. As a result, massive accumulation of cisplatin in lysosomes might trigger TFEB activation in resistant cells. In contrast, lysosomal platinum in sensitive cells apparently does not reach an amount that is sufficient to activate TFEB. Interestingly, multiple types of human cancers exhibit higher TFEB activity [43,44,45,46], which is associated with multidrug resistance, an aggressive phenotype, and poor prognosis [47,48,49,50]. 

Apart from the lysosomal stress, TFEB signaling networks might be directly regulated by metals like Pt and Cu. Recent studies show that TFEB nuclear translocation might be caused by elevated copper levels [27]. Considering that copper operates as an allosteric modulator of some kinases [3,5], it is tempting to speculate that Cu^+^ and highly similar Pt^+^ ions could (directly or indirectly) impact the activities of mTOR kinase and calcineurin phosphatase, which regulate TFEB phosphorylation and, hence, its nuclear translocation. 

Finally, Pt might regulate TFEB activity at the level of its interaction with the chromatin of tumor cells. By ChIP, we found that endogenous TFEB preferentially binds upstream and to the first intron CLEAR sites within the ATP7B promoter, although all 3 CLEAR binding sites are functionally relevant in increasing ATP7B transcriptional activity, as demonstrated by the luciferase reporter data. Notably, TFEB direct binding occurs only upon Pt exposure, while no significant binding has been found in the untreated chromatin condition, meaning that Pt might be able to change chromatin conformation and its accessibility to transcription factors, which contribute to the gained resistance [51]. Studies confirm that metals such as Cu and Pt are able to modify the methylation and histone chromatin profile of ATP7B acting at the interface between genetics and the environment. Indeed, studies on chemoresistant ovarian cancer revealed methionine and associated pathways of glutathione synthesis and polyamine biosynthesis to be the most significantly altered [52]. Therefore, it is possible that Pt-induced demethylation changes the chromatin conformation, thereby facilitating TFEB access to the *ATP7B* CLEAR sites and, thereby, allowing TFEB to accelerate *ATP7B* transcription in IGROV-CP20 cells. As a consequence, higher amounts of ATP7B might be used by resistant cells to counteract incoming Pt drugs. 

It is highly likely that TFEB-mediated transactivation of ATP7B is used as a Pt defense mechanism by different tumor cells. Our own findings suggest that inhibitor of TFEB nuclear translocation increases cisplatin toxicity in another ovarian cancer cell line A2780-CP20, whose resistance to Pt involves elevated ATP7B expression [12,39]. Therefore, this mechanism could be shared by different types of ovarian tumors with high ATP7B levels. Ovarian cancer is the second leading cause of death from gynecological malignancies worldwide [53], and the current standard care requires platinum-based chemotherapies in a very substantial cohort of patients. Considering that over 70% of patients will ultimately relapse and develop chemoresistance [54], Pt resistance represents a serious concern. In this context, ATP7B overexpression emerges as a well-documented risk factor, which predicts poor outcomes of Pt-based chemotherapy in patients [7,8]. Therefore, targeting the TFEB/ATP7B transcription axis should help to address this issue.

We also believe that the role of TFEB in Pt resistance expands beyond upregulation of ATP7B and might involve several additional mechanisms. TFEB has been shown to counteract Pt and Cu toxicity through activation of autophagy and biogenesis of lysosomes that sequester these metals [26,27]. A recent study shows that TFEB suppression in tongue squamous cell carcinoma line reduces Pt tolerance [26]. This study also suggests that TFEB-mediated lysosome biogenesis rather than autophagy contributes to Pt detoxification in these cells. However, how TFEB activity translates into the ability of lysosomes to neutralize Pt drugs remains unclear. Upregulation of ATP7B provides an example of how this may happen, while other mechanisms might include transactivation of the other lysosomal genes regulating Pt transport across the lysosomal membrane, sequestration of Pt into exosomes or lysosomal exocytosis [11,18,21,24]. Thus, further efforts should be conducted to understand the TFEB-mediated mechanism of Pt-resistance and their therapeutic relevance.

## Data Availability

The data presented in this study are available on request from the corresponding author.
